# Machine learning molecular dynamics simulations toward exploration of high-temperature properties of nuclear fuel materials: case study of thorium dioxide

**DOI:** 10.1038/s41598-022-13869-9

**Published:** 2022-06-13

**Authors:** Keita Kobayashi, Masahiko Okumura, Hiroki Nakamura, Mitsuhiro Itakura, Masahiko Machida, Michael W. D. Cooper

**Affiliations:** 1 CCSE, Japan Atomic Energy Agency, Kashiwa, Chiba 277-0871 Japan; 2 Materials Science and Technology Division, Los Alamos National Laboratory, P.O. Box 1663, Los Alamos, NM 87545 USA

**Keywords:** Condensed-matter physics, Theory and computation, Atomic and molecular physics

## Abstract

Predicting materials properties of nuclear fuel compounds is a challenging task in materials science. Their thermodynamical behaviors around and above the operational temperature are essential for the design of nuclear reactors. However, they are not easy to measure, because the target temperature range is too high to perform various standard experiments safely and accurately. Moreover, theoretical methods such as first-principles calculations also suffer from the computational limitations in calculating thermodynamical properties due to their high calculation-costs and complicated electronic structures stemming from *f*-orbital occupations of valence electrons in actinide elements. Here, we demonstrate, for the first time, machine-learning molecular-dynamics to theoretically explore high-temperature thermodynamical properties of a nuclear fuel material, thorium dioxide. The target compound satisfies first-principles calculation accuracy because *f*-electron occupation coincidentally diminishes and the scheme meets sampling sufficiency because it works at the computational cost of classical molecular-dynamics levels. We prepare a set of training data using first-principles molecular dynamics with small number of atoms, which cannot directly evaluate thermodynamical properties but captures essential atomistic dynamics at the high temperature range. Then, we construct a machine-learning molecular-dynamics potential and carry out large-scale molecular-dynamics calculations. Consequently, we successfully access two kinds of thermodynamic phase transitions, namely the melting and the anomalous $\lambda$ transition induced by large diffusions of oxygen atoms. Furthermore, we quantitatively reproduce various experimental data in the best agreement manner by selecting a density functional scheme known as SCAN. Our results suggest that the present scale-up simulation-scheme using machine-learning techniques opens up a new pathway on theoretical studies of not only nuclear fuel compounds, but also a variety of similar materials that contain both heavy and light elements, like thorium dioxide.

## Introduction

Thorium has attracted much attention as a potential nuclear fuel^1,2^. Thorium is now estimated to be three to four times more abundant in nature than uranium the shortage of which might become a concern in the coming future. Moreover, its nuclear-fuel material form, thorium dioxide, is chemically more stable than the uranium-based counterpart^3^. Owing to the above primary and other several advantages, thorium dioxide is considered to be a promising candidate fuel material in next-generation nuclear reactors.

The detailed information of nuclear fuel materials in a high temperature range around its melting point is prerequisite for not only design of reactors but also nuclear safety. However, it is generally difficult to measure physical properties in such a high temperature range due to limitation of durability of experiment instruments and resultant concern about safety. It is also difficult to maintain the stoichiometry of some fuel compounds at such high temperatures (e.g. PuO${}_{2}$). Therefore, the experimental data of thermal properties of thorium dioxide as well as other fuel materials has not been still accumulated sufficiently in the temperature region. Thus, a theoretical approach accurately examining material properties in atomic-levels, i.e., molecular dynamics (MD) simulation has been intensively employed as an alternative important tool to complement insufficient experimental data^4,5^.

Calculations of thermal properties through MD simulation require large-size and long-time runs in order to achieve statistical-mechanically reliable accuracy. Then, classical MD using empirical atomic force fields has been a primary scheme among various ones, because it allows statistically convergent properties to be obtained with reasonable computational costs. Indeed, several authors^6–13^ studied thermal properties of thorium compounds using classical MD. However, it should be noted that the obtained results strongly depend on the empirical parameters of the force field. This fact clearly indicates that careful development of atomic potentials is crucial for reliability of the calculated thermal properties. Then, their comparative studies among possible potential candidates are essential together with experimental results^14^.

An alternative way to calculate thermal properties of materials is using first-principles calculations based on density functional theory (DFT)^15^. Its ab-initio style has made a great impact on atomic-level simulation studies because of their non-empirical modeling. However, first-principles calculations for thorium dioxide have been so far limited only in a few literatures^16–19^. In the previous study^19^, two of the authors have explicitly shown that first-principles molecular dynamics (FPMD) simulations provide reliable data of thermal properties of thorium dioxides in the high temperature range, but the system size and averaging time were severely restricted due to its huge computational costs.

In the last decade, machine learning has been used as a tool to construct atomic potentials. The machine learning techniques are utilized to train potential energy surfaces (PES) with first principles accuracy by interpolation among a large number of reference data obtained by first principles calculations^20–23^. One of promising machine learning approaches is a method using artificial neural networks (ANN) proposed by Behler and Parrinello^20,22^. We call the ANN Behler-Parrinello neural networks (BPNN) throughout this paper. In contrast to empirical atomic force fields, BPNN is not based on any physical modeling but have a large number of adjustable parameters. The rich flexibility in BPNN enables us to make PES of which accuracy is comparable to those calculated from first-principles.

Generally, machine learning molecular dynamics (MLMD) using BPNN is expected to access thermal properties with first-principles accuracy even in unavailable large system sizes and long average times for FPMD. Actually, using the advantage of MLMD, structural phase transitions have been successfully examined by MLMD^24^. However, these cases demand not so large system size because the phase transition among different solid phases can be well captured with periodic boundary conditions. On the contrary, mixture of phases including liquids and/or gases require large systems to evaluate physical processes. In this paper, using MLMD with BPNN, we evaluate thermal properties of thorium dioxide, as an example of nuclear fuels, with first principles accuracy in a wide temperature range, whose upper limit is beyond the melting point.

Thorium dioxide has a fluorite structure with space group Fm${\bar{3}}$m, in which the 4a and 8c positions are occupied by thorium and oxygen ions, respectively. The lattice constant at room temperature is 5.592 Å^25^, and the melting point is 3651 K^26^. In addition, another kind of phase transition was reported below the melting point as a pre-melting phase transition^27,28^. The transition is expected as a diffuse transition of 8c position elements, which is called the Bredig transition^29^ or simply $\lambda$-transition. Recently, anomalous oxygen dynamics around the transition has been intensively studied using classical MD motivated by a close resemblance to anomalous atomic dynamics widely seen in glass forming systems^30,31^. The electron structure of thorium dioxide is rather simple. The localized *f*-electrons of actinide oxides usually influence thermal properties at high temperature due to their strongly-correlated features as seen in the cases of plutonium dioxide^32^. On the other hand, Th cations in thorium dioxide principally loose all electrons in the outer-*f*-shell, resulting no *f*-occupation. Then, the thermal properties of thorium dioxide are regarded to be well described by only the movement of atoms by the valence electrons. However, the ionic interaction is actually affected by atomic-charge polarizations and emergent interactions. In DFT calculation, their descriptions depend on the choice of exchange-correlation (XC) functional. Therefore, we train BPNN using DFT reference data sets based on different typical XC functionals. Using MLMD with several BPNNs, we conduct a systematic study on the high-temperature thermal properties of thorium dioxide.

## Methods

Vienna ab initio Simulation Package (VASP)^33,34^ is used for obtaining reference data sets for BPNN. In all calculations, the projector-augmented wave method^35^ is employed, and 500 eV energy cutoff is chosen. In this study, we use three types of XC functionals: the local density approximation (LDA) in the parametrization of Ceperly and Alder^36^, the generalized gradient approximation of Perdew–Burke–Ernzerhof for solids (GGA-PBEsol)^37^, and the strongly constrained and appropriately normed (SCAN) meta-GGA XC functional^38^.

First, we perform FPMD *NPT* simulations with PBEsol functional from 300 to 5000 K with a 100 K temperature step. The combination of the Langevin thermostat and Parrinello–Rahman barostat is adopted to generate the NPT ensemble. The time step and simulation total time at each temperature are 2 fs and 16 ps, respectively. Potashnikov et al.^14^ pointed out that the smallest cell size to capture the Bredig transition in MD simulations is $3 \times 3\times 3$ of the unit cell. Thus, we also choose $3 \times 3\times 3$ supercell of thorium dioxide (324 atoms) and only $\Gamma$ point is used as a k-point mesh. We randomly pick up 9000 snapshots of the MD simulations as the reference data based on PBEsol. For creating the reference data based on LDA and SCAN functionals, we randomly select 3000 structures from the dataset based on PBEsol, and evaluate the energies and forces of the 3000 configurations by DFT calculations with LDA and SCAN. The 3000 structures recalculated by DFT with LDA and SCAN are used as the reference data sets for BPNNs based on LDA and SCAN. Furthermore, the adaptive learning scheme^39–43^ is used to improve the quality of the reference datasets. In this scheme, we create two BPNNs with different initial weights and conduct MLMD simulations to generate various structures of ThO$_{2}$. Next, we select structures with large force differences between the outputs of the two BPNNs from the generated structures. Finally, we re-evaluate the energies and forces for the selected structures by DFT and add these to the reference data. As a result, the total numbers of the reference data based on LDA and SCAN XC functionals are 7749 and 7007 structures, respectively.

We use the n2p2 code^44^ for training BPNN. In BPNN, a local environment of each atom with a cutoff radius $R_{\mathrm{c}}$ is encoded to descriptor vectors. We adopt the following type-2 and type-4 symmetry functions^22^ as the descriptors of the distances and the angles of atoms, respectively, i.e.,

1 $$\begin{aligned} G_{i}^{(2)}= & {} \sum _{j} e^{-\eta ^{(2)}(R_{ij}-R_{\mathrm{s}})^2}f_{\mathrm{c}}(R_{ij}) \,, \end{aligned}$$

2 $$\begin{aligned} G_{i}^{(4)}= & {} 2^{1-\xi }\sum _{j\ne i}\sum _{k\ne i,j} \left( 1+\lambda \cos \theta _{ijk}\right) ^{\xi } e^{-\eta ^{(4)}(R_{ij}^{2}+R_{ik}^{2}+R_{jk}^{2})} f_\mathrm{c}(R_{ij})f_{\mathrm{c}}(R_{ik})f_{\mathrm{c}}(R_{jk}), \end{aligned}$$

with the cutoff function

3 $$f_{{\text{c}}} (R) = \left\{ {\begin{array}{*{20}l} {0.5\cos \left( {\frac{{\pi R}}{{R_{{\text{c}}} }} + 1} \right)} \hfill & {{\text{for}}\quad R \le R_{{\text{c}}} } \hfill \\ 0 \hfill & {{\text{for}}\quad R_{{\text{c}}} < R} \hfill \\ \end{array} } \right.,$$

where $R_{ij}$ is the distance between the *i*-th and *j*-th atoms, $\theta _{ijk}$ is the angle formed by line segments between the *i*-th and *j*-th atoms and the *i*-th and *k*-th ones. The cutoff radius $R_{\mathrm{c}}$ for $G_i^{(2)}$ and $G_i^{(4)}$ are taken as 8.0 Å and 6.5 Å, respectively. We choose the parameter $R_\mathrm {s}= 0.0$, and the other parameters were selected by CUR decomposition^45^. First, we creat symmetry functions with a total $N_{\mathrm{SF}}=240$ dimension and construct $N_{\mathrm{sample}} \times N_{\mathrm{SF}}$ feature matrix *X*, where each column vector consist of the symmetry function of the corresponding sample. Then, we perform a CUR decomposition for the feature matrix *X* and select the symmetry functions that satisfies the following criteria: $||\varvec{X}-\varvec{CUR}||_{\mathrm{F}}/||\varvec{X}||_{\mathrm{F}} \le 10^{-4}$, where $||\cdot ||_{\mathrm{F}}$ denote the Frobenius norm. The detailed lists of the selected symmetry functions are shown in the Supplemental Materials. Using a dataset consisting of a selected descriptor vector and corresponding first-principles energy and forces, BPNNs are trained. We use two hidden layers with hyperbolic tangent activation functions with 30 nodes. The multistream Kalman filter method^46^ is adopted as an optimizer for BPNN. 90% of the reference data is assigned to training data and the remaining 10% as test data. We construct three machine learning potentials using data generated by DFT with the LDA, PBEsol and SCAN XC functionals, which are referred to as BPNN-LDA, BPNN-PBEsol and BPNN-SCAN, respectively.

In this paper, all MD simulations are carried out by LAMMPS^47^. *NPT* simulations are performed with Nosè–Hoover thermostat and barostat relaxation times being 0.1 ps and 0.5 ps, respectively.

## Results

### Accuracy of machine learning potentials

Table 1 summarizes the root mean square errors (RMSE) of energy and force for the training and test data. The RMSEs of the present BPNNs for the reference data are below 2.4 meV/atom for the reference energies and 8.3$\times 10^{-2}$ eV/Å for the forces, which are comparable with the typical RMSEs in previous studies^20,43,48–50^. In order to test the accuracy of BPNNs, we compare physical quantities obtained by DFTs, BPNNs, empirical atomic potentials, and experiments data. In this paper, we choose BD08^8^ and the Cooper, Rushton and Grimes (CRG)^9^ empirical atomic potentials for comparison with DFTs and BPNNs. BD08 atomic potential is a relatively simple pairwise potential consisting of the Coulombic and the Buckingham potentials^51^. On the other hand, CRG atomic potential includes many-body EAM-type potential^52^ in addition to the pairwise potentials.

**Table 1:** RMSE of BPNN-LDA, BPNN-PBEsol and BPNN-SCAN for the training and test data.

	BPNN-LDA		BPNN-PBEsol		BPNN-SCAN	
	Training	Test	Training	Test	Training	Test
Energy (meV/atom)	2.085	2.108	2.297	2.357	1.207	1.264
Force ($10^{-2}$ eV/Å)	5.705	6.551	7.906	8.239	5.812	5.769

**Figure 1:**
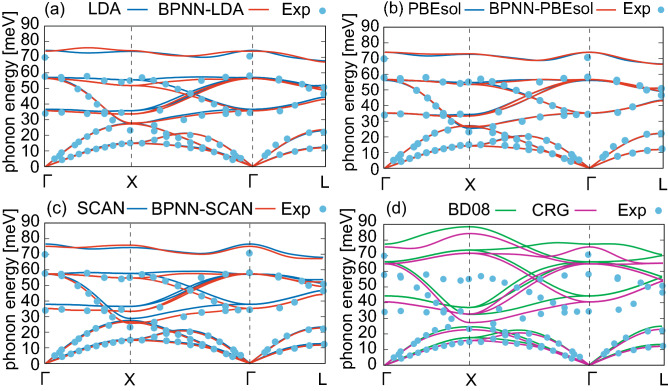
Phonon dispersion curves for ThO$_{2}$. (**a–c**) Phonon dispersion obtained by (BPNN-)LDA, (BPNN-)PBEsol, and (BPNN-)SCAN where black and red line are the results computed by DFT and BPNN, respectively. (**d**) Are the results obtained by BD08 (green line) and CRG potential (purple line). Circle dots in (**a–d**) represent the experimental data.^57^

We compute the lattice constant, elastic properties, and phonon dispersion curves at zero temperature using DFTs, BPNNs, and empirical atomic potentials. The elastic constants are calculated by a numerical differential of the stress tensors with respect to finite strains. The phonon bands within the harmonic approximation are obtained using Phonopy^58^ with the finite difference method. In phonon calculations, to treat long range interaction of macroscopic electric field induced by polarization of atomic displacement near $\Gamma$ point, we add non-analytical correction by dipole-dipole interaction to dynamical matrix^59,60^ (see also the Supplementary Materials). The computed results are shown in Table 2 and Fig. 1. The lattice constant and elastic data of BPNN calculations agree well with those by DFT ones. The results computed by (BPNN-)PBEsol and (BPNN-)SCAN show similar lattice and elastic constants, which also agree with the experiment ones^25,53–55^, whereas (BPNN-)LDA slightly underestimates the lattice constant. Comparing the results obtained by DFTs, BPNNs and empirical atomic potentials, the lattice constant and elastic data calculated by BD08 and CRG potentials seem to be more accurate than the results obtained by DFTs and BPNNs. Note that this is not necessarily surprising given that the empirical potentials are fitted to these experimental properties. The phonon dispersion curves calculated by BPNNs are also in good agreement with the curves obtained by DFTs, as shown in Fig. 1a,b. Furthermore, DFTs and BPNNs reproduce the experimental data^57^ almost completely. Especially, we note that optical modes in the phonon dispersion curves calculated by the empirical atomic potentials show large deviation from the experimental data as shown in Fig. 1c though the results of DFTs and BPNNs are almost perfect in these modes.

**Table 2:** Lattice constant and elastic properties of ThO${}_{2}$ obtained by DFTs, BPNNs, emprical potentials, and experiments (Exp.).

	DFT			BPNN			Empirical potential		Exp.
	LDA	PBEsol	SCAN	LDA	PBEsol	SCAN	BD08	CRG	
Lattice constant (Å)	5.529	5.576	5.598	5.531 (0.04)	5.565 (0.20)	5.610 (0.21)	5.600	5.580	5.592^25^
$C_{11}$ (GPa)	385.6	373.0	378.9	374.0 (3.01)	370.8 (0.59)	348.7 (7.97)	367	352.3	367.0^53^
$C_{12}$ (GPa)	130.2	120.7	118.2	142.2 (9.22)	122.7 (1.66)	124.2 (5.08)	106	113.4	106.0^53^
$C_{44}$ (GPa)	82.0	80.7	83.7	75.9 (7.44)	73.9 (8.43)	74.3 (11.23)	95	71.7	79.6^53^
Bulk modulus (GPa)	215.3	204.8	205.1	219.5 (1.95)	199.0 (2.83)	205.4 (0.15)	193	193.0	193.0^53^
									$195.0\pm 2$ ^54^
									$198.0\pm 2$ ^55^
Shear modulus (GPa)	98.0	96.6	100.0	90.0 (8.16)	91.1 (5.69)	87.7 ( 12.30)	107.9	88.1	95.6–100.6^53^
									$103\pm 2$ ^56^

The round brackets $(\cdot )$ in the BPNN columns represent the percentage errors of the BPNN results against the DFT results.

So far, we have validated the BPNN potentials using static calculations. However, the validations for dynamical calculation are also required, since inappropriate BPNN potentials sometimes cause unstable MLMD and result in structural collapse with a long simulation period, especially at high temperatures^61^. On the other hand, MLMDs using the present BPNNs show good stability in long-period NVE simulations as shown in the supplementary materials. Thus, BPNNs trained in the present study enable us to conduct MLMD simulations over a long period with no anomalies.

### Thermal expansion, enthalpy and specific heat capacity

In the above, BPNNs are found to have accuracy comparable to DFTs. Next, we apply MLMD to large-scale simulations, which are difficult to perform by FPMD. The present MLMD is about several hundred thousand times faster than FPMD (see the computational efficiency of BPNNs summarized in the Supplementary Materials). MLMD enables us to easily evaluate the thermal properties of ThO$_2$ with the almost FPMD accuracy.

Here, we focus on a linear thermal expansion (LTE)

4 $$\begin{aligned} \mathrm{LTE} = \frac{L(T)-L(300\, \text{K})}{L(300 \, \text{K})}, \end{aligned}$$

and an averaged coefficient of linear thermal expansion (ACLTE)

5 $$\begin{aligned} \mathrm{ACLTE} = \frac{1}{T_{2}-T_{1}}\int _{T_{1}}^{T_{2}}\frac{1}{L(T)}\frac{\partial L(T)}{\partial T}dT = \frac{\log L(T_2)-\log L(T_1)}{T_{2}-T_{1}}, \end{aligned}$$

where *L*(*T*) is a lattice constant at temperature *T*. The values of the lattice constant at 300 K are listed in Table 3. We also calculate enthalpy *H*(*T*) and molar specific heat capacity at constant pressure, which is a critical quantity for discussion of the $\lambda$-transition,

6 $$\begin{aligned} C_{p} = \frac{1}{n}\frac{\partial H(T)}{\partial T}, \end{aligned}$$

where *n* is the amount of substance in moles. Calculations of these properties require large system size and long averaging time to avoid the finite size effects. In addition, computations of the heat capacity and ACLTE require numerical measurements at a large number of temperature points with a tiny temperature step elevation for smooth numerical differentials. Therefore, it is difficult to evaluate these quantities by FPMD. Then, we perform MLMD *NPT* simulations using $6 \times 6 \times 6$ supercell (2592 atoms) and totally 200 ps run per 10 K temperature step. The $6 \times 6 \times 6$ supercell is large enough to neglect finite size effects and to evaluate the thermal properties of ThO$_{2}$ as shown in the supplementary material. Moreover, in order to smooth the curves of heat capacity and ACLTE, we average their values over the interval of ± 100 K twice as performed in reference^14^ (see also the Supplementary Material). For the comparison, we also calculate the thermal properties using classical MD with BD08 and CRG empirical potentials.

**Table 3:** The lattice constant *L*(*T*) at 300 K, the averaged coefficient of linear thermal expansion (ACLTE), the onset temperature of heat capacity anomaly $T_{\alpha }$, and the $\lambda$-peak temperature $T_{\mathrm{c}}$ are summarized.

	BPNN-LDA	BPNN-PBEsol	BPNN-SCAN	BD08	CRG	Exp.
*L*(300K)	5.545	5.580	5.624	5.616	5.595	5.592^25^
ACLTE (10$^{-6}$ K$^{-1}$)	9.95	10.65	9.71	10.84	10.05	9.5^62^, 9.67^63^, 11.07^26^
$T_{\alpha }$ (K)	2360	2350	2460	2760	2450	
$T_{\mathrm{c}}$ (K)	3040	2980	3200	3440	2930	2950^27^, 3090^28^

**Figure 2:**
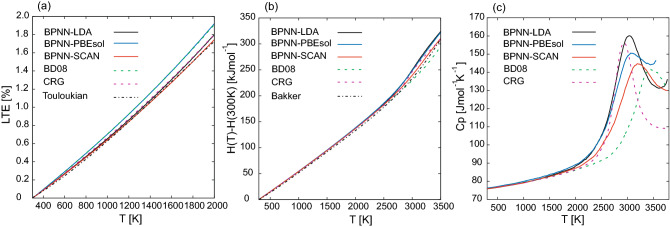
(**a–c**) Temperature dependence of the LTE, the enthalpy and the molar specific heat capacity. The solid lines are the results obtained by MLMD with BPNN-LDA (black line), BPNN-PBEsol (blue line), and BPNN-SCAN (red line), respectively, whereas the dashed lines are the results computed by MD with BD08 (green line) and CRG potential (purple line). The black dashed-dot line in (**a,b**) represent the Touloukian^62^ and the Bekker fitting^64^ equations for the experiment.

Figure 2 shows the temperature dependence of LTE, enthalpy, and specific heat capacity $C_{p}$. Among the LTEs obtained by MLMDs and classical MDs as shown in Fig. 2a, the results computed by BPNN-SCAN show the best agreement with the Touloukian fitting of the experimental results, which is available up to 2000 K. The ACLTEs in the range from 300 to 1600K are listed in Table 3. The ACLTE computed by BPNN-SCAN also shows good agreement with the experimental data $9.5 \times 10^{-6}$ K$^{-1}$ from Momin (298–1600 K)^62^ and $9.67 \times 10^{-6}$ K$^{-1}$ by Rodriguez (293–2273 K)^63^.

In enthalpy calculation as shown in Fig. 2a, the results computed by BPNN-SCAN and CRG potential provide close values to the Bekker fitting of the experiment^64^ over the entire temperature range. All computed enthalpies give close values in the low-temperature range, but show different behavior in the high-temperature range where the specific heat anomaly emerges as shown in Fig. 2b. The onset temperature of the specific heat anomaly can be characterized as $T_{\alpha }$, which is defined as the temperature giving the minimum value of $C_{p}/T$^65^. $T_{\alpha }$ are ordered as BPNN-LDA, BPNN-PBEsol < CRG, BPNN-SCAN < BD08 as listed in Table 3. The peak position of $C_{p}$ obtained by BPNNs and CRG potential are in good agreement with the experimental results 2950 K^27^ and 3090 K^28^ reported as the Bredig transition temperature.

### Oxygen diffusion and defect concentration

**Figure 3:**
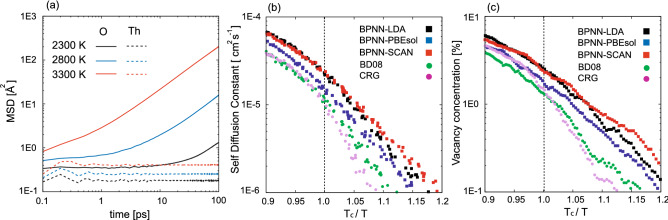
(**a**) MSD obtained by MLMD with BPNN-SCAN. (**b**) Temperature dependence of the self-diffusion constant for oxygen. (**c**) Temperature dependence of the vacancy concentration.

We evaluate the mean square displacements (MSD) defined as

7 $$\begin{aligned} \mathrm{MSD}(t) = \frac{1}{N_{\alpha }}\sum _{i}^{N_{\alpha }}|\varvec{r}_{i}(t)-\varvec{r}_{i}(0)|^{2}, \end{aligned}$$

where $\varvec{r}_{i}(t)$ is the position of the *i*-th atom at time *t* and $N_{\alpha }$ is the total number of $\alpha$ atoms ($\alpha$ is Th or O). The employed system size and total time-step are the same as the cases computing the thermal expansion and the molar specific heat capacity. We conduct *NVE* simulations with 1 fs time-step at various temperatures using the volumes previously calculated in *NPT* ensemble. Figure 3a shows the MSD computed by MLMD with BPNN-SCAN. In the previous study using FPMD^19^, accurate evaluations of MSD could not be performed due to its high computational costs. In contrast, MLMD easily overcomes such a limitation, and then sufficiently long simulations allow us to detect the diffusive regime even in the temperature region below $T_{\mathrm{c}}$ =3200 K (see MSD of oxygen at 2300 K in Fig. 3a). It should also be mentioned that Th shows vibrational motions below and above the transition temperature. We evaluate the self-diffusion constant *D* for the oxygen atoms from the slope of MSD in the range 25 to 100 ps. Figure 3b shows the temperature dependence of the self-diffusion constant for the oxygen atom. In all MLMD and clasical MD simulations, one can find the bending of the Arrhenius plot of the self-diffusion constant above $T_{\mathrm{c}}$. The deviation of the self-diffusion constant from Arrhenius law was experimentally reported in PbF$_{2}$^66,67^, which belongs to the fluorite-type structure like ThO$_{2}$. We calculate the activation energy of diffusion below $T_{\mathrm{c}}$ using the Arrhenius relation, $D = D_{0}\exp (-E_{\mathrm{AE}}/kT)$. Table 4 shows the values of the activation energy obtained by MLMDs and empirical MDs. All BPNNs give similar activation energies, which are lower than those obtained by the empirical atomic potential.

The specific heat anomaly and dynamics of anions have been investigated related to the disorders in fluorite, and defect cluster models have been proposed so far^68,69^. In this study, we focus on the vacancy concentration of oxygen in the regular site. In an ideal fluorite structure, one oxygen exits within a cube with vertices: (0, 0, 0), (0, 0, 1/2), (0, 1/2, 0), (1/2, 0, 0), (0, 1/2, 1/2), (1/2, 1/2, 0), (1/2, 0, 1/2), and (1/2, 1/2, 1/2). Thus, we count a number of cubes not including oxygen from MD trajectories and define the vacancy concentration as the ratio of the empty cubes. As with the self-diffusion constant for oxygen, the temperature dependence of the oxygen vacancy concentrations obeys the Arrhenius law below $T_{\mathrm{c}}$, and the Arrhenius plots bent downwards above $T_{\mathrm{c}}$ as shown in Fig. 3c. These results indicate that the origin of the lambda transition and anion dynamics are closely related to the defect formation. The vacancy concentration of oxygen obtained by MLMDs and classical MDs are from 1 to 3% at $T_{\mathrm{c}}$ and are within 10% above $T_{\mathrm{c}}$. The low defect concentration of ThO$_{2}$ below $T_{\mathrm{c}}$ is consistent with the experimental results of fluorite materials^70–72^.

**Table 4:** Activation energy of diffusion below $T_{\mathrm{c}}$ and vacancy concentration at $T_{\mathrm{c}}$.

	BPNN-LDA	BPNN-PBEsol	BPNN-SCAN	BD08	CRG
Activation energy below $T_{\mathrm{c}}$ (eV)	4.851	4.446	4.400	7.049	6.696
Vacancy concentration at $T_{\mathrm{c}}$ (%)	2.5	1.7	2.3	1.3	1.5

### Melting temperature

**Figure 4:**
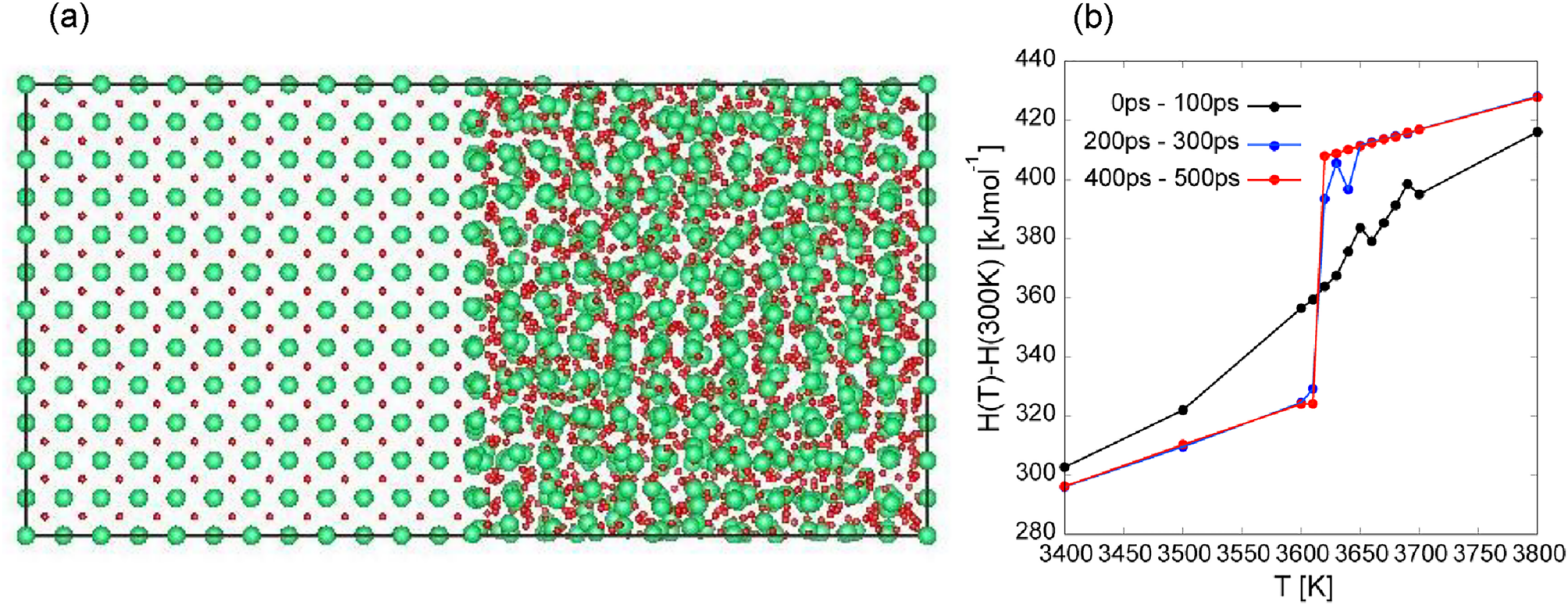
(**a**) Configuration of $6 \times 6 \times 12$ supercell including solid (left) and liquid phase (right). (**b**) Temperature dependence of the enthalpy obtained by the two-phase simulation approach using MLMD with BPNN-SCAN. The blue filled circles and the open green squares and red circles represent the enthalpies calculated in the periods as 0 ps to 100 ps, 200 ps to 300 ps and 400 ps to 500 ps, respectively.

The melting temperature of thorium dioxide can be determined by the so-called two-phase simulation approach. A $6 \times 6 \times 12$ supercell (5184 atoms) including both solid and liquid phases is prepared as an initial configuration as shown in Fig. 4a. MLMD *NPT* simulations are performed from 3000 to 4000 K, in which the simulation time is taken over 500 ps. Figure 4b shows averaged enthalpy calculated by BPNN-SCAN in the periods from 0 to 100 ps, 200 to 300 ps, and 400 to 500 ps, respectively. From Fig. 4b, we can confirm that the enthalpy jump at 3620 K and the melting point evaluated by MLMD with BPNN-SCAN lies between 3610–3620 K. The melting temperatures evaluated by MLMDs and classical MDs are also summarized in Table 5. BPNN-SCAN closely reproduce the experimental melting point (3651 K) while BPNN-LDA give somewhat lower melting point (3450–3460 K) and BPNN-PBEsol significantly underestimate it as 3250–3260 K. The choice of XC functional seems to be sensitive in evaluating accurate melting temperature. In calculation using empirical potentials, CRG potential provides accurate melting temperature, whereas BD08 potential overestimates it as shown in Table 5.

**Table 5:** The melting points evaluated by two phase simulations.

	BPNN-LDA	BPNN-PBEsol	BPNN-SCAN	BD08	CRG	Exp.
$T_{\mathrm{m}}$ (K)	3450–3460	3250–3260	3610–3620	3810–3820	3640–3650	3651^27^

## Conclusion

MLMD simulations using BPNN were extensively performed to evaluate the thermal properties of a fuel material, thorium dioxide. In this paper, we made three types of BPNNs based on DFT reference data with LDA, PBEsol, and SCAN XC-functionals. We confirmed that the constructed BPNNs have close accuracy with DFTs through the comparisons of lattice constant, elastic properties, and phonon dispersion, which also well agree with the experimental data. Moreover, large-size and long-run simulations being inaccessible for FPMD were successfully performed by using MLMD. Through the systematic studies for thermal properties of thorium dioxide, the BPNN-SCAN especially gave the closest results to the experimental fitting data for the thermal expansion and the enthalpy, and well reproduced the experimental melting temperature. Therefore, we judge that the BPNN-SCAN provides reasonable results for all testable experimental data. The BPNN-LDA and -PBEsol also showed reasonable results for several thermal properties of thorium dioxide, but they underestimated the experimental melting temperature. Comparing the XC functionals employed in this study, SCAN functional includes intermediate-range attractive dispersion interaction^73^, which is absent in other standard DFT functionals. Actually, it has been reported that the inclusion of dispersion interaction improves not only the description of the structural properties^73,74^ but also melting temperature and liquid properties^75^. Therefore, it turns out that the intermediate-range attractive dispersion interaction of the BPNN-SCAN leads to a correct description of the thermal properties of thorium dioxide.

In this study, we focused on the perfect bulk system and did not explicitly include the defect structures of thorium dioxide in the reference data. However, the present BPNNs can predict the defect formation energies for various defect structures with a certain degree of accuracy as shown in the Supplementary Material. The inclusion of liquid structures and the structures with oxygen diffusion at high temperatures is considered to make BPNNs possible to describe the various defect structures. Therefore, by adding some DFT data on defect structures to the reference data, the present BPNNs is expected to be applied to damage analysis under high radiation fields intrinsic to nuclear fuels. Further extension of the BPNNs to describe irradiation damage and surface state of thorium dioxide is an important future work.

In conclusion, we confirmed that the present MLMD is a powerful computation tool to explore high-temperature materials properties of thorium dioxide, one of oxide fuel compounds, with keeping first-principles accuracy. The state-of-the-art simulation scheme is further expected to find out the detailed physics of some unsolved phenomena just below the melting transition from microscopic levels. In principle, MLMD can be applicable for the calculation of thermal properties of other actinide dioxides. A key issue will be the construction of the reference dataset based on XC functionals describing strongly correlated *f*-electrons correctly. For example, to reproduce the insulator phase of other actinide dioxides, it is essential to use more sophisticated methods such as DFT+U or hybrid functional approach^76–79^. Once the reference dataset based on proper DFT methods is created, MLMD can capture high-temperature thermodynamical features in the first-principles accuracy as shown in the present study.

## Supplementary Information

Supplementary Information.

## Data Availability

The machine learning potentials (BPNNs) created in this study are included in the Supplementary Information files. The datasets generated during the current study are available from the corresponding author on reasonable request.
