# Supplementary material for: Machine learning molecular dynamics simulations toward exploration of high-temperature properties of nuclear fuel materials: case study of thorium dioxide
Source: Sci Rep. 2022 Jun 13;12:9808. doi: 10.1038/s41598-022-13869-9 (PMC9192752; doi:10.1038/s41598-022-13869-9)
Supplement: Supplementary file 1 — Supplementary Information. [file 41598_2022_13869_MOESM1_ESM.pdf]

Supplementary material for " Machine learning molecular dynamics simulations toward exploration of high-temperature properties of nuclear fuel materials: thorium dioxide case"

Keita Kobayashi[a] Masahiko Okumura[a], Hiroki Nakamura[a], Mitsuhiro Itakura[a], Masahiko Machida[a], and Michael W.D. Cooper[b]

1 The parameters of symmetry functions and parity plot

In this section, we show the parameters of symmetry functions employed in the main text and the parity plots of the energies and forces given by BPNN as compared to DFT reference values.

We generated symmetry functions with the combination of parameters: $\eta^{(2)} = (0.01562,$ 0.02242, 0.03217, 0.04616, 0.06623, 0.09503, 0.13636, 0.19566, 0.28075, 0.40283, 0.57802, 0.82938, 1.19001, 1.70757, 2.45014, 3.51563), $\eta^{(4)} =$ (0.02367, 0.03751, 0.05945, 0.09423, 0.14934, 0.23669, 0.37512, 0.59453, 0.94227, 1.49339, 2.36686), $\lambda = (-1, 1)$, and $\xi = (1, 4, 8)$. The cutoff radius R_c for $G_i^{(2)}$ and $G_i^{(4)}$ were taken as 8.0 Å and 6.5 Å, respectively. These are typical values employed in the previous works about machine learning potentials for an ionic-covalent system[1,2]. The number of symmetry functions for thorium and oxygen atoms becomes (240,240). Using a CUR decomposition, we selected (49,40), (40,31), and (49,39) symmetry functions for BPNN-LDA, BPNN-PBEsol, and BPNN-SCAN, respectively. The detailed parameters of symmetry functions for BPNN-LDA, BPNN-PBEsol, and BPNN-SCAN are listed in Table S1, S2, and S3

Table S1: The parameters for the symmetry function of BPNN-LDA.

Type of symmetry function	Combination	$\eta^{(2),(4)}$	λ	ξ	R_c
type-2	O-O	0.01562	–	–	8.0
type-2	O-O	0.04616	–	–	8.0
type-2	O-O	0.09503	–	–	8.0
type-2	O-O	0.1957	–	–	8.0
type-2	O-O	0.2807	–	–	8.0
type-2	O-O	0.578	–	–	8.0
type-2	O-Th	0.01562	–	–	8.0
type-2	O-Th	0.04616	–	–	8.0
type-2	O-Th	0.1364	–	–	8.0
type-2	O-Th	0.2807	–	–	8.0

[a] Center for Computational Science and e-Systems, Japan Atomic Energy Agency, Kashiwa, Chiba 277–0871, Japan
[b] Materials Science and Technology Division, Los Alamos National Laboratory, P.O. Box 1663, Los Alamos, NM, 87545, USA

type-2	O-Th	0.4028	–	–	8.0
type-2	O-Th	0.8294	–	–	8.0
type-2	Th-O	0.01562	–	–	8.0
type-2	Th-O	0.04616	–	–	8.0
type-2	Th-O	0.09503	–	–	8.0
type-2	Th-O	0.1957	–	–	8.0
type-2	Th-O	0.4028	–	–	8.0
type-2	Th-O	0.8294	–	–	8.0
type-2	Th-Th	0.01562	–	–	8.0
type-2	Th-Th	0.03217	–	–	8.0
type-2	Th-Th	0.06623	–	–	8.0
type-2	Th-Th	0.1957	–	–	8.0
type-2	Th-Th	0.4028	–	–	8.0
type-4	O-O-O	0.02367	-1	1.0	6.5
type-4	O-O-O	0.02367	1	1.0	6.5
type-4	O-O-O	0.02367	-1	4.0	6.5
type-4	O-O-O	0.02367	-1	8.0	6.5
type-4	O-O-O	0.02367	1	8.0	6.5
type-4	O-O-O	0.03751	1	4.0	6.5
type-4	O-O-O	0.05945	-1	1.0	6.5
type-4	O-O-O	0.05945	1	1.0	6.5
type-4	O-O-O	0.05945	-1	4.0	6.5
type-4	O-O-O	0.05945	1	8.0	6.5
type-4	O-O-O	0.09423	1	4.0	6.5
type-4	O-O-O	0.1493	1	1.0	6.5
type-4	O-O-Th	0.02367	-1	1.0	6.5
type-4	O-O-Th	0.02367	1	1.0	6.5
type-4	O-O-Th	0.02367	-1	4.0	6.5
type-4	O-O-Th	0.02367	1	4.0	6.5
type-4	O-O-Th	0.02367	-1	8.0	6.5
type-4	O-O-Th	0.02367	1	8.0	6.5
type-4	O-O-Th	0.03751	1	1.0	6.5
type-4	O-O-Th	0.03751	1	4.0	6.5
type-4	O-O-Th	0.05945	-1	1.0	6.5
type-4	O-O-Th	0.05945	1	1.0	6.5
type-4	O-O-Th	0.05945	-1	4.0	6.5
type-4	O-O-Th	0.05945	1	8.0	6.5
type-4	O-O-Th	0.09423	1	4.0	6.5
type-4	O-O-Th	0.1493	1	1.0	6.5
type-4	O-Th-Th	0.02367	-1	1.0	6.5
type-4	O-Th-Th	0.02367	1	1.0	6.5
type-4	O-Th-Th	0.02367	-1	4.0	6.5
type-4	O-Th-Th	0.02367	1	4.0	6.5
type-4	O-Th-Th	0.02367	-1	8.0	6.5

type-4	O-Th-Th	0.02367	1	8.0	6.5
type-4	O-Th-Th	0.05945	-1	1.0	6.5
type-4	O-Th-Th	0.05945	1	1.0	6.5
type-4	O-Th-Th	0.05945	-1	4.0	6.5
type-4	O-Th-Th	0.05945	1	8.0	6.5
type-4	O-Th-Th	0.1493	1	1.0	6.5
type-4	Th-O-O	0.02367	-1	1.0	6.5
type-4	Th-O-O	0.02367	1	1.0	6.5
type-4	Th-O-O	0.02367	-1	4.0	6.5
type-4	Th-O-O	0.02367	-1	8.0	6.5
type-4	Th-O-O	0.02367	1	8.0	6.5
type-4	Th-O-O	0.03751	1	1.0	6.5
type-4	Th-O-O	0.03751	1	4.0	6.5
type-4	Th-O-O	0.05945	-1	1.0	6.5
type-4	Th-O-O	0.05945	1	1.0	6.5
type-4	Th-O-O	0.05945	-1	4.0	6.5
type-4	Th-O-O	0.05945	-1	8.0	6.5
type-4	Th-O-O	0.05945	1	8.0	6.5
type-4	Th-O-O	0.09423	1	4.0	6.5
type-4	Th-O-O	0.1493	-1	1.0	6.5
type-4	Th-O-O	0.1493	1	1.0	6.5
type-4	Th-O-Th	0.02367	-1	1.0	6.5
type-4	Th-O-Th	0.02367	1	1.0	6.5
type-4	Th-O-Th	0.02367	-1	4.0	6.5
type-4	Th-O-Th	0.02367	-1	8.0	6.5
type-4	Th-O-Th	0.02367	1	8.0	6.5
type-4	Th-O-Th	0.03751	1	4.0	6.5
type-4	Th-O-Th	0.05945	-1	1.0	6.5
type-4	Th-O-Th	0.05945	1	1.0	6.5
type-4	Th-O-Th	0.05945	1	8.0	6.5
type-4	Th-O-Th	0.1493	1	1.0	6.5
type-4	Th-Th-Th	0.02367	-1	1.0	6.5
type-4	Th-Th-Th	0.02367	1	1.0	6.5
type-4	Th-Th-Th	0.02367	1	4.0	6.5
type-4	Th-Th-Th	0.05945	1	1.0	6.5

Table S2: The parameters for the symmetry function of BPNN-BPEsol.

Type of symmetry function	Combination	$\eta^{(2),(4)}$	λ	ξ	R_c
type-2	O-O	0.01562	–	–	8.0
type-2	O-O	0.03217	–	–	8.0
type-2	O-O	0.06623	–	–	8.0
type-2	O-O	0.1364	–	–	8.0

type-2	O-O	0.2807	–	–	8.0
type-2	O-O	0.578	–	–	8.0
type-2	O-Th	0.01562	–	–	8.0
type-2	O-Th	0.04616	–	–	8.0
type-2	O-Th	0.09503	–	–	8.0
type-2	O-Th	0.1957	–	–	8.0
type-2	O-Th	0.4028	–	–	8.0
type-2	Th-O	0.01562	–	–	8.0
type-2	Th-O	0.04616	–	–	8.0
type-2	Th-O	0.09503	–	–	8.0
type-2	Th-O	0.1957	–	–	8.0
type-2	Th-O	0.4028	–	–	8.0
type-2	Th-O	0.8294	–	–	8.0
type-2	Th-Th	0.01562	–	–	8.0
type-2	Th-Th	0.03217	–	–	8.0
type-2	Th-Th	0.06623	–	–	8.0
type-2	Th-Th	0.1957	–	–	8.0
type-4	O-O-O	0.02367	-1	1.0	6.5
type-4	O-O-O	0.02367	1	1.0	6.5
type-4	O-O-O	0.02367	-1	4.0	6.5
type-4	O-O-O	0.02367	1	4.0	6.5
type-4	O-O-O	0.02367	-1	8.0	6.5
type-4	O-O-O	0.02367	1	8.0	6.5
type-4	O-O-O	0.03751	1	4.0	6.5
type-4	O-O-O	0.05945	-1	1.0	6.5
type-4	O-O-O	0.05945	1	1.0	6.5
type-4	O-O-O	0.1493	1	1.0	6.5
type-4	O-O-Th	0.02367	-1	1.0	6.5
type-4	O-O-Th	0.02367	1	1.0	6.5
type-4	O-O-Th	0.02367	-1	4.0	6.5
type-4	O-O-Th	0.02367	1	4.0	6.5
type-4	O-O-Th	0.02367	-1	8.0	6.5
type-4	O-O-Th	0.02367	1	8.0	6.5
type-4	O-O-Th	0.05945	-1	1.0	6.5
type-4	O-O-Th	0.05945	1	1.0	6.5
type-4	O-O-Th	0.05945	1	4.0	6.5
type-4	O-O-Th	0.05945	1	8.0	6.5
type-4	O-O-Th	0.1493	1	1.0	6.5
type-4	O-Th-Th	0.02367	-1	1.0	6.5
type-4	O-Th-Th	0.02367	1	1.0	6.5
type-4	O-Th-Th	0.02367	-1	4.0	6.5
type-4	O-Th-Th	0.02367	1	4.0	6.5
type-4	O-Th-Th	0.02367	-1	8.0	6.5
type-4	O-Th-Th	0.02367	1	8.0	6.5

type-4	O-Th-Th	0.05945	-1	1.0	6.5
type-4	O-Th-Th	0.05945	1	1.0	6.5
type-4	Th-O-O	0.02367	-1	1.0	6.5
type-4	Th-O-O	0.02367	1	1.0	6.5
type-4	Th-O-O	0.02367	-1	4.0	6.5
type-4	Th-O-O	0.02367	-1	8.0	6.5
type-4	Th-O-O	0.02367	1	8.0	6.5
type-4	Th-O-O	0.03751	1	4.0	6.5
type-4	Th-O-O	0.05945	-1	1.0	6.5
type-4	Th-O-O	0.05945	1	1.0	6.5
type-4	Th-O-O	0.05945	-1	4.0	6.5
type-4	Th-O-O	0.05945	1	8.0	6.5
type-4	Th-O-O	0.1493	1	1.0	6.5
type-4	Th-O-Th	0.02367	-1	1.0	6.5
type-4	Th-O-Th	0.02367	1	1.0	6.5
type-4	Th-O-Th	0.02367	-1	4.0	6.5
type-4	Th-O-Th	0.02367	1	4.0	6.5
type-4	Th-O-Th	0.02367	1	8.0	6.5
type-4	Th-O-Th	0.05945	1	1.0	6.5
type-4	Th-O-Th	0.05945	1	8.0	6.5
type-4	Th-O-Th	0.09423	1	1.0	6.5
type-4	Th-Th-Th	0.02367	1	1.0	6.5
type-4	Th-Th-Th	0.02367	1	4.0	6.5

Table S3: The parameters for the symmetry function of BPNN-SCAN.

Type of symmetry function	Combination	$\eta^{(2),(4)}$	λ	ξ	R_c
type-2	O-O	0.01562	–	–	8.0
type-2	O-O	0.04616	–	–	8.0
type-2	O-O	0.09503	–	–	8.0
type-2	O-O	0.1957	–	–	8.0
type-2	O-O	0.2807	–	–	8.0
type-2	O-O	0.578	–	–	8.0
type-2	O-O	1.19	–	–	8.0
type-2	O-Th	0.01562	–	–	8.0
type-2	O-Th	0.04616	–	–	8.0
type-2	O-Th	0.1364	–	–	8.0
type-2	O-Th	0.2807	–	–	8.0
type-2	O-Th	0.4028	–	–	8.0
type-2	O-Th	0.8294	–	–	8.0
type-2	Th-O	0.01562	–	–	8.0
type-2	Th-O	0.04616	–	–	8.0
type-2	Th-O	0.09503	–	–	8.0

type-2	Th-O	0.1957	–	–	8.0
type-2	Th-O	0.4028	–	–	8.0
type-2	Th-O	0.8294	–	–	8.0
type-2	Th-Th	0.01562	–	–	8.0
type-2	Th-Th	0.03217	–	–	8.0
type-2	Th-Th	0.06623	–	–	8.0
type-2	Th-Th	0.1957	–	–	8.0
type-2	Th-Th	0.4028	–	–	8.0
type-4	O-O-O	0.02367	-1	1.0	6.5
type-4	O-O-O	0.02367	1	1.0	6.5
type-4	O-O-O	0.02367	-1	4.0	6.5
type-4	O-O-O	0.02367	-1	8.0	6.5
type-4	O-O-O	0.02367	1	8.0	6.5
type-4	O-O-O	0.03751	1	4.0	6.5
type-4	O-O-O	0.05945	-1	1.0	6.5
type-4	O-O-O	0.05945	1	1.0	6.5
type-4	O-O-O	0.05945	-1	4.0	6.5
type-4	O-O-O	0.05945	1	8.0	6.5
type-4	O-O-O	0.09423	1	4.0	6.5
type-4	O-O-O	0.1493	1	1.0	6.5
type-4	O-O-Th	0.02367	-1	1.0	6.5
type-4	O-O-Th	0.02367	1	1.0	6.5
type-4	O-O-Th	0.02367	-1	4.0	6.5
type-4	O-O-Th	0.02367	1	4.0	6.5
type-4	O-O-Th	0.02367	-1	8.0	6.5
type-4	O-O-Th	0.02367	1	8.0	6.5
type-4	O-O-Th	0.03751	1	1.0	6.5
type-4	O-O-Th	0.03751	1	4.0	6.5
type-4	O-O-Th	0.05945	-1	1.0	6.5
type-4	O-O-Th	0.05945	1	1.0	6.5
type-4	O-O-Th	0.05945	-1	4.0	6.5
type-4	O-O-Th	0.05945	1	8.0	6.5
type-4	O-O-Th	0.09423	1	4.0	6.5
type-4	O-O-Th	0.1493	1	1.0	6.5
type-4	O-Th-Th	0.02367	-1	1.0	6.5
type-4	O-Th-Th	0.02367	1	1.0	6.5
type-4	O-Th-Th	0.02367	-1	4.0	6.5
type-4	O-Th-Th	0.02367	1	4.0	6.5
type-4	O-Th-Th	0.02367	-1	8.0	6.5
type-4	O-Th-Th	0.02367	1	8.0	6.5
type-4	O-Th-Th	0.05945	-1	1.0	6.5
type-4	O-Th-Th	0.05945	1	1.0	6.5
type-4	O-Th-Th	0.05945	1	4.0	6.5
type-4	O-Th-Th	0.05945	-1	8.0	6.5

type-4	Th-O-O	0.02367	-1	1.0	6.5
type-4	Th-O-O	0.02367	1	1.0	6.5
type-4	Th-O-O	0.02367	-1	4.0	6.5
type-4	Th-O-O	0.02367	-1	8.0	6.5
type-4	Th-O-O	0.02367	1	8.0	6.5
type-4	Th-O-O	0.03751	1	4.0	6.5
type-4	Th-O-O	0.05945	-1	1.0	6.5
type-4	Th-O-O	0.05945	1	1.0	6.5
type-4	Th-O-O	0.05945	-1	4.0	6.5
type-4	Th-O-O	0.05945	-1	8.0	6.5
type-4	Th-O-O	0.05945	1	8.0	6.5
type-4	Th-O-O	0.09423	1	4.0	6.5
type-4	Th-O-O	0.1493	-1	1.0	6.5
type-4	Th-O-O	0.1493	1	1.0	6.5
type-4	Th-O-Th	0.02367	-1	1.0	6.5
type-4	Th-O-Th	0.02367	1	1.0	6.5
type-4	Th-O-Th	0.02367	-1	4.0	6.5
type-4	Th-O-Th	0.02367	-1	8.0	6.5
type-4	Th-O-Th	0.02367	1	8.0	6.5
type-4	Th-O-Th	0.03751	1	4.0	6.5
type-4	Th-O-Th	0.05945	-1	1.0	6.5
type-4	Th-O-Th	0.05945	1	1.0	6.5
type-4	Th-O-Th	0.05945	1	8.0	6.5
type-4	Th-O-Th	0.09423	1	1.0	6.5
type-4	Th-Th-Th	0.02367	-1	1.0	6.5
type-4	Th-Th-Th	0.02367	1	1.0	6.5
type-4	Th-Th-Th	0.02367	1	4.0	6.5
type-4	Th-Th-Th	0.05945	1	1.0	6.5

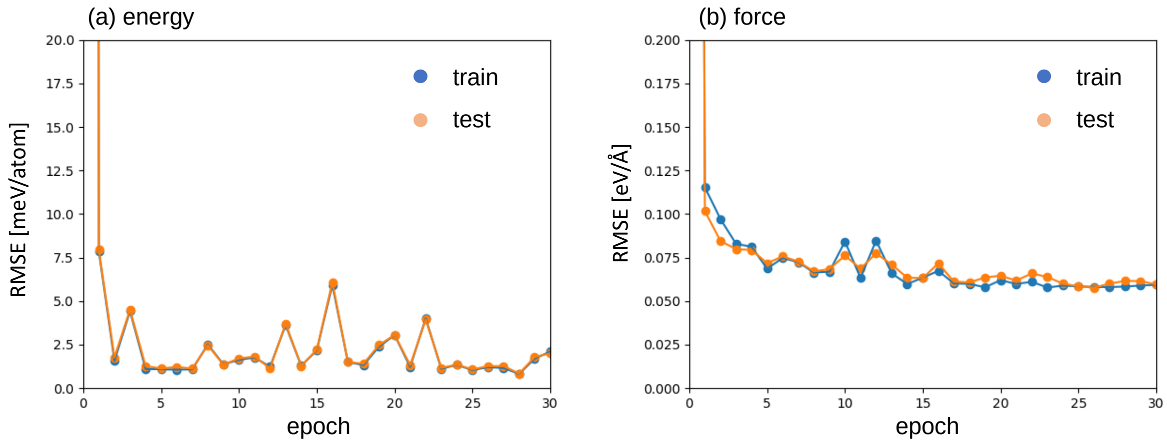

Fig. S1: (a) and (b) show the root mean square error (RMSE) of BPNN-SCAN for energy (eV/atom) and force (eV/Å) as a function of the number of epochs. Blue and orange filled circles represent the RMSE for the training and test data, respectively.

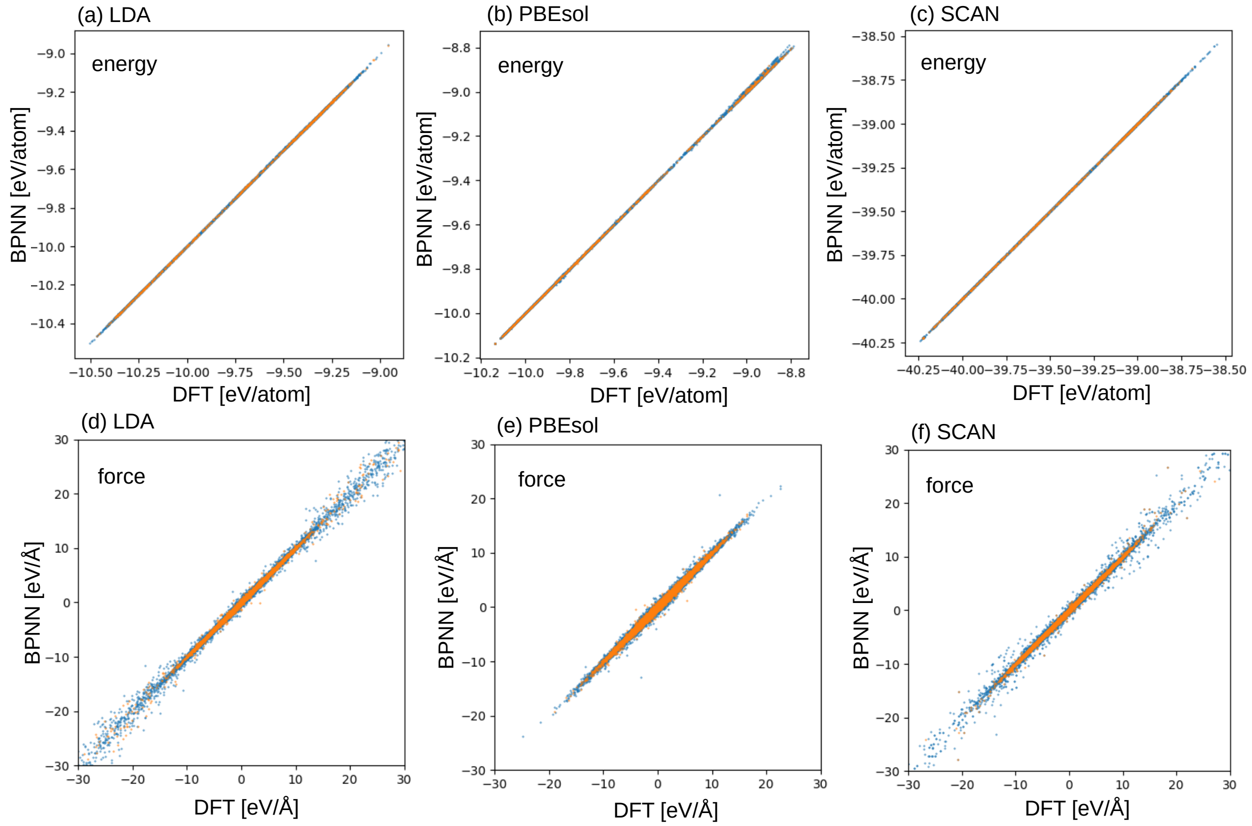

Fig. S2: The parity plots of the energies (eV/atom) and the forces (eV/Å) for the training (blue filled circles) and test (orange filled circles) data. Horizontal and vertical axes represent the values given by DFT and BPNN, respectively.

Using the selected descriptor vector listed in Table S1, S2, and S3, we trained BPNNs using the multistream Kalman filter method[3,4] with 30 epochs. 90% of the reference data was assigned to training data and the remaining 10% to test data. Both root mean square errors (RMSE) decreased rapidly in a few epochs as shown in figure S1. The RMSE of energies continued to show small oscillations even as the epoch progressed, but the RMSE of forces converged to an almost constant value. We selected the BPNN with the smallest RMSE of energies for the test data within 30 epochs. The resulting RMSEs were summarized in table 1 in the main text. The parity plots of the energies and forces obtained by BPNNs are shown in figure S2. We can find that a good linear relationship is realized for both energies and forces obtained by DFTs and BPNNs. Comparing BPNN-LDA, BPNN-PBEsol, and BPNN-SCAN, the ranges of forces included in the reference data of BPNN-LDA and BPNN-SCAN were broader than that of BPNN-PBEsol. The difference is considered due to the method used to generate the reference data set. The reference data set of BPNN-PBEsol was generated by only FPMD simulation, whereas these of BPNN-LDA and BPNN-SCAN were mainly created by adiabatic learning scheme. The structures including large forces acting on atoms do not frequently appear in FPMD simulation. On the other hand, the active learning scheme explores structures that are not sufficiently contained in the reference data. Therefore, the range of forces in the reference data of BPNN-LDA and BPNN-SCAN became broader than that of BPNN-PBEsol.

2 Validation of BPNNs for dynamical calculation

The validation of BPNNs in the main text focused on static calculations. Therefore, in this section, we show the validation of BPNNs for dynamical calculations.

2.1 Stability of MLMDs at high temperature

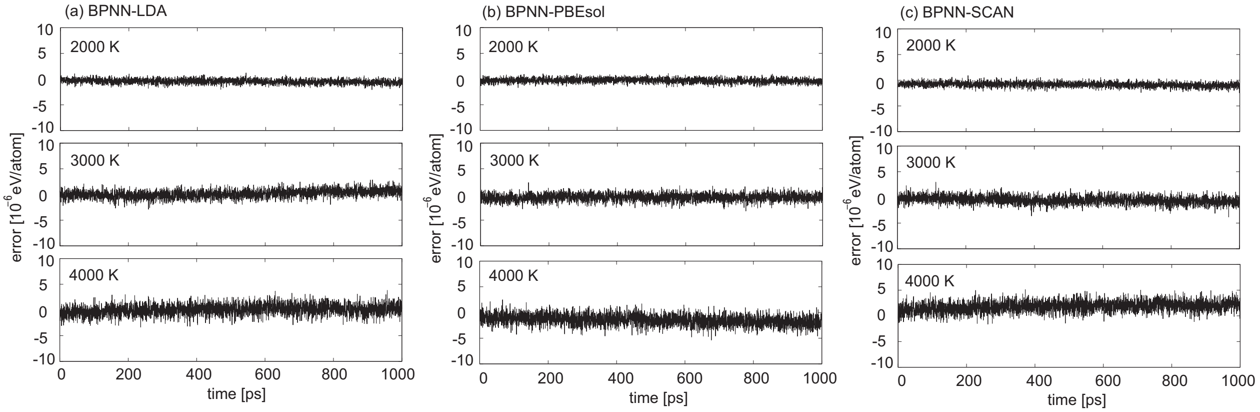

Fig. S3: (a), (b), and (c) show the errors in conservation of total energy per atom computed by MLMD *NVE* simulations with BPNN-LDA, BPNN-PBEsol, and BPNN-SCAN, respectively.

First, we show the stability of MLMD for dynamical calculation. MD simulation with BPNN trained with inappropriate descriptors, hyperparameters, and reference data sometimes be-

comes unstable and show structural collapse with a long simulation period, especially at high temperatures[5]. Therefore, we checked the stability of MLMD with a long simulation period at high temperatures. We conducted MLMD *NVE* simulations at 2000, 3000, and 4000 K. The number of atoms was 2592, and the total simulation time was 1000 ps with 1 fs step size. To verify the energy conservation of MLMD *NVE* simulations, we show the time evolution of the error for total energy in figure S3, which is defined as

$$\text{error}(t) = \frac{E(t) - E(0)}{N}\,, \tag{1}$$

where $E(t)$ is the total energy at time t and N is the number of atoms. The errors obtained by MLMDs with BPNN-LDA, BPNN-PBEsol, and BPNN-SCAN were within $\pm 5 \times 10^{-6}$ eV per atom, and the total energies computed by MLMDs were well conserved over a long period with no anomalies. These results denote that BPNNs can be smoothly fitted to the potential energy surface of DFTs.

2.2 Comparison of the mean square displacements obtained by FPMD and MLMD

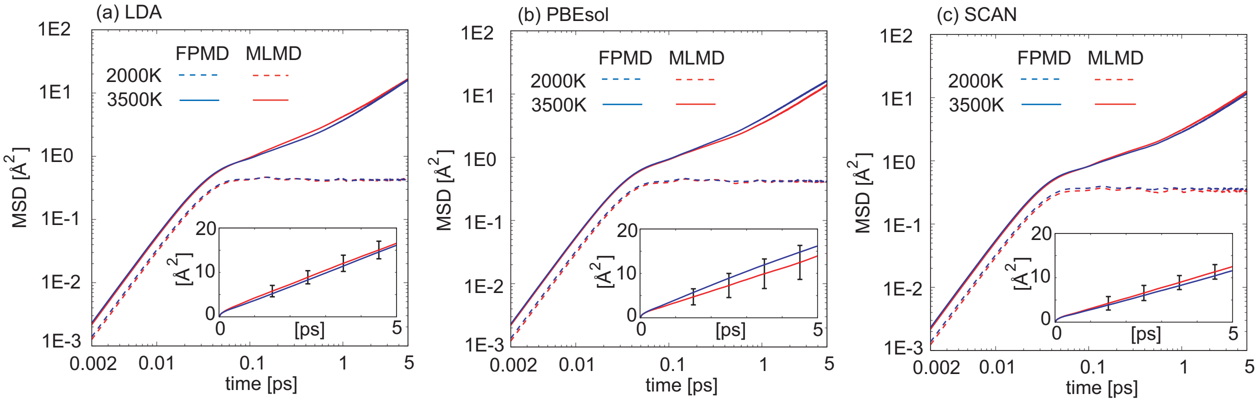

Fig. S4: The mean square displacement (MSD) computed by FPMD and MLMD simulations at 2000 and 3500 K. (a), (b), and (c) are the results with (BPNN-)LDA, (BPNN-)PBEsol, and (BPNN-)SCAN in the log-log plot, respectively. The insets of figures are the MSD with the error bars at 3500 K .

In this subsection, we compare the mean square displacement (MSD) for short trajectories obtained by FPMDs and MLMDs. We conducted FPMD and MLMD *NVT* simulations with $2 \times 2 \times 2$ supercell (96 atoms). The lattice constant was fixed to 5.8 Å. Time step size and total simulation time of *NVT* simulation were 2 fs and 25 ps, respectively. A Nosè-Hoover thermostat were applied for the simulations with relaxation times of 0.08 ps. We calculated ensemble average of MSD from $t = 0$ to 5 ps time period as

$$\text{MSD}(t) = \sum_{i}^{N} \sum_{t_0}^{T_0} \frac{1}{NT_0} |\boldsymbol{r}_i(t + t_0) - \boldsymbol{r}_i(t_0)|^2\,, \tag{2}$$

where N is the number of oxygen atoms and $T_0 = 20$ ps. Figure S4 shows the MSD obtained by FPMD and MLMD at 2000 and 3500 K. We can find that the results obtained by FPMD and MLMD are in good agreement.

2.3 Comparison of the entalpy and lattice constant obtained by FPMD and MLMD

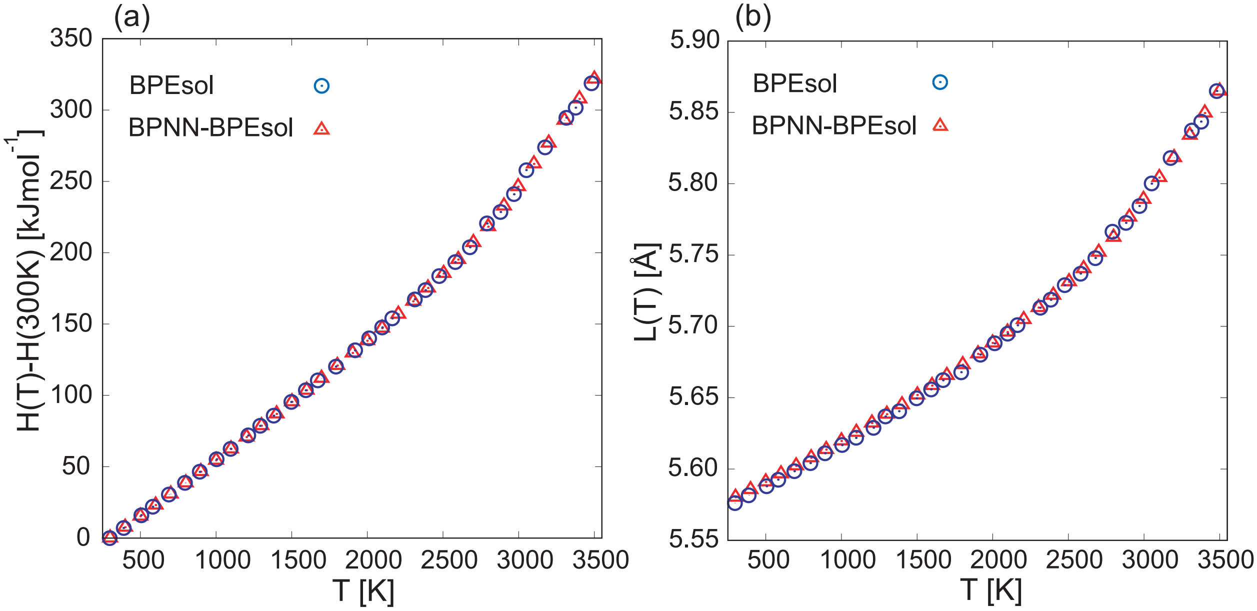

Fig. S5: (a) Temperature dependence of the enthalpy $H(T)$. (b) Temperature dependence of the lattice constant $L(T)$. The green circles and purble triangles represent the results obtained by FPMD with PBEsol and MLMD with BPNN-PBEsol, respectively.

This subsection shows the validation of thermophysical properties obtained by BPNN. Since all reference data about BPNN-PBEsol was generated by FPMD at various temperatures, thermophysical properties obtained by FPMD and MLMD based on PBEsol functional can be compared. Here, we compared the temperature dependence of the enthalpy $H(T)$ and the lattice constant $L(T)$ from 300 to 3500 K using FPMD and MLMD NPT. For both methods, we use $3 \times 3 \times 3$ supercells (324 atoms). The time step size and simulation total time of FPMD at each temperature were 2 fs and 16 ps, respectively. MLMD was conducted with 2 fs time step and a total 100 ps simulation time. The results shown in figure S5 reveal that the temperature dependence of enthalpy $H(T)$ and lattice constant $L(T)$ obtained by MLMD shows a good agreement with that computed by FPMD.

3 Computational efficiency of BPNNs

Table S4: The elapsed time per MD step of FPMDs and MLMDs based on LDA, PBEsol, and SCAN with the different number of atoms.

Number of atoms	96	324	768	1500	2592	4116	6144
FPMD-LDA [s]	9.75	163.64	—	—	—	—	—
FPMD-PBEsol [s]	9.45	156.52	—	—	—	—	—
FPMD-SCAN [s]	22.69	720.00	—	—	—	—	—
BPNN-LDA [10^{-2}s]	0.09	0.17	0.27	0.50	0.78	1.21	1.67
BPNN-PBEsol [10^{-2}s]	0.07	0.14	0.24	0.44	0.66	1.03	1.43
BPNN-SCAN [10^{-2}s]	0.08	0.17	0.27	0.53	0.74	1.19	1.63

In this section, we show the computational efficiency of the present MLMD. The NVT simulations at 3500 K were conducted for ThO_2 with the different number of atoms. We used 240 CPU cores on the supercomputing system HPE SGI8600 supporting Intel Xeon Gold 6242R (3.1 GHz, 40 core) in Japan Atomic Energy Agency. The FPMD simulations were limited up to 324 atoms due to their high computational costs. The elapsed times of FPMDs and MLMDs per MD step are summarized in Table S4. In the case of 96 atoms, MLMDs were about tens of thousands of times faster than FPMD. Furthermore, MLMDs with 324 atoms were about several hundred thousand times faster than FPMDs with 324 atoms.

4 The method of smoothing the curve of specific heat capacity

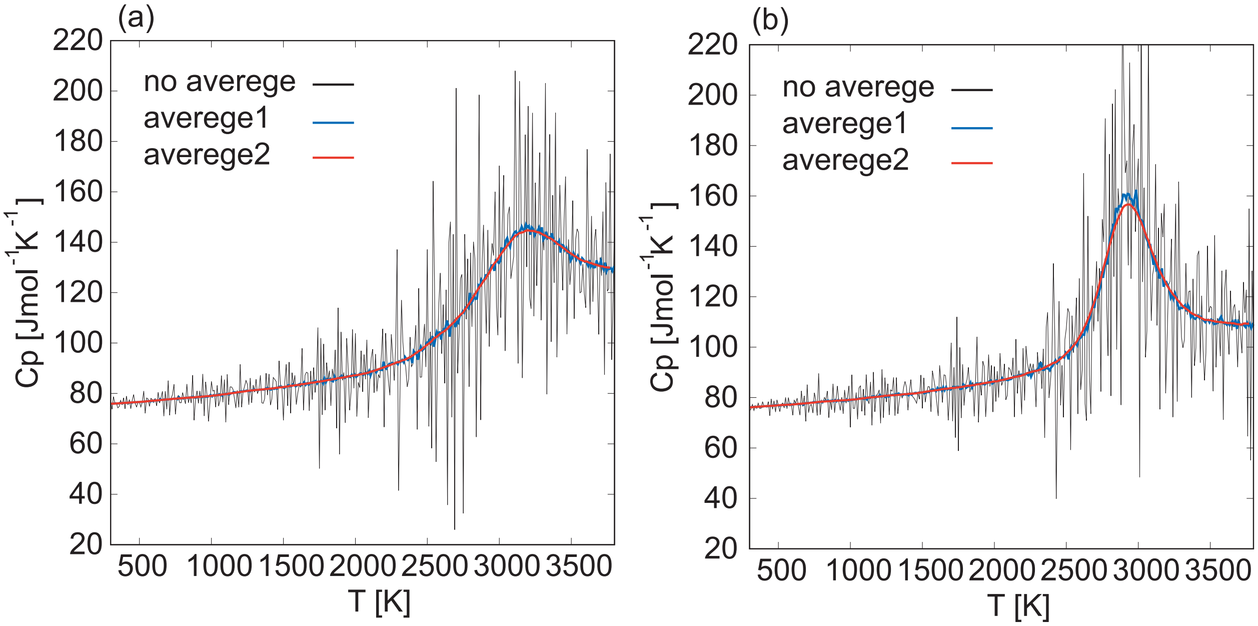

Fig. S6: (a) and (b): The temperature dependence of specific heat capacity obtained from the numerical differentiation of the enthalpy, which are computed by NPT simulation with BPNN-SCAN and CRG potential. Black lines are the results without smoothing the specific heat capacity curve. Blue and red lines are the results smoothed by averaging the curve (Black lines) over the interval of ±100 K once and twice, respectively.

In this section, we show the detail of smoothing the curve of heat capacity. We performed MLMD NPT simulations with 2592 atoms and total 200 ps run per 10 K temperature step. The molar specific heat capacity was obtained from the numerical differentiation of the enthalpy

$$C_p^{(0)}(T = NdT) = \frac{1}{n}\frac{H((N+1)dT) - H((N-1)dT)}{dT}\,, \tag{3}$$

where n is the amount of substance in moles, H is the enthalpy and dT is temperature step ($dT = 10$). In order to smooth the curve of specific heat capacity calculated by Eq (3), we averaged specific heat capacity over the interval of ±100 K twice[6] as

$$C_p^{(1)}(T = NdT) = \sum_{i=N-M}^{N+M} \frac{C_p^{(0)}(idT)}{M}\,, \tag{4}$$

$$C_p^{(2)}(T = NdT) = \sum_{i=N-M}^{N+M} \frac{C_p^{(1)}(idT)}{M}\,. \tag{5}$$

where $M = 10$. Figure S6 shows the curves of specific heat with no averaging operation, averaging operation once and averaging operation twice.

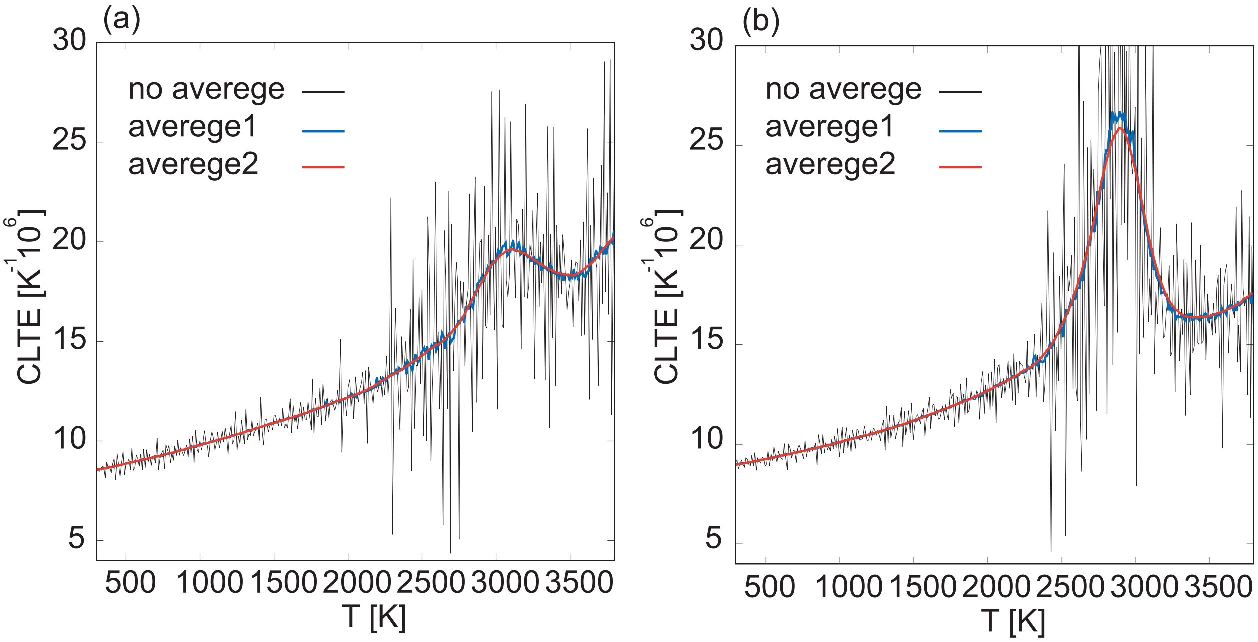

Fig. S7: (a) and (b): The temperature dependence of CLTE obtained from the numerical differentiation of the lattice constant, which are computed by *NPT* simulation with BPNN-SCAN and CRG potential. Black lines are the results without smoothing the CLTE curve. Bule and red lines are the results smoothed by averaging the curve (Black lines) over the interval of ±100 K once and twice, respectively.

In figure S7, we also show the curves of the coefficient of linear thermal expansion (CLTE) defined as

$$\mathrm{CLTE} = \frac{1}{L(T)}\frac{dL(T)}{dT}\,, \tag{6}$$

where $L(T)$ is the lattice constant at temperature T. The smoothing of the curve was done in the same way as done in the specific heat capacity curve. The ACLTE defined in the main text is equivalent to the average of CLTE from 300 to 1600 K.

5 Size dependence of specific heat capacity and coefficient of linear thermal expansion

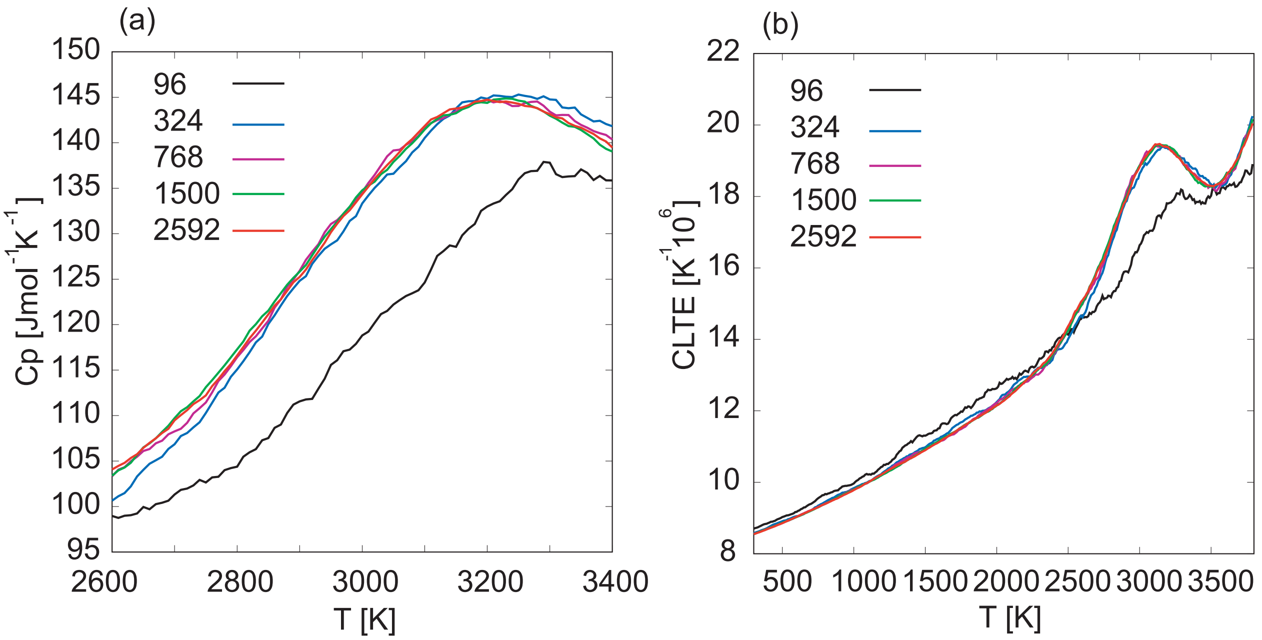

Fig. S8: (a) and (b): The size dependence of specific heat capacity around peak temperature and CLTE obtained by MLMD with BPNN-SCAN. The system sizes were changed as 96 (black line), 324 (blue line), 768 (purple line), 1500 (green line), and 2592 (red line) atoms, respectively.

Figure S8.(a) shows the size dependence of specific heat capacity by changing the number of atoms in the range from 96 ($2 \times 2 \times 2$ cell) to 2592 ($6 \times 6 \times 6$ cell). The temperatures of λ-peak became lower with increasing system size, and the shapes of λ-peak were almost unchanged above 1500 atoms. Therefore, the system size with 2592 atoms is considered sufficient to neglect finite size effects for calculating thermal properties using MLMD. The size dependence of CLTE was also small above 768 atoms ($4 \times 4 \times 4$ cell) as shown in figure S8(b). The size dependence for the thermal properties of ThO_2 using MLMD was similar to the previous study about that of UO_2 using empirical force field[6].

6 Defect formation energy obtained by DFTs and BPNNs

Table S5: The defect formation energies computed by DFTs and BPNNs. Sch*, OFP*, and ThFP denote the formation energy of Schottky, oxygen Frenkel pair, and thorium Frenkel pair defects, respectively. The round brackets $(\cdot)$ represent the percentage errors of the BPNN results against the DFT results

type of defect	Sch1	Sch2	Sch3	OFP1	OFP2	ThFP
DFT-LDA [eV]	5.69	5.76	6.26	4.55	4.10	13.23
BPNN-LDA [eV]	5.65	5.72	6.23	4.22	3.77	13.82
	(0.70)	(0.70)	(0.48)	(7.26)	(8.05)	(4.46)
DFT-PBEsol [eV]	5.12	5.17	5.66	4.38	3.93	12.31
BPNN-PBEsol [eV]	5.5	5.55	6.06	4.17	3.78	16.46
	(7.42)	(7.35)	(7.07)	(4.79)	(3.82)	(33.71)
DFT-SCAN [eV]	5.42	5.48	6.00	4.55	4.05	13.16
BPNN-SCAN [eV]	5.51	5.55	6.07	4.17	3.72	14.56
	(1.66)	(1.28)	(1.17)	(8.35)	(8.15)	(10.64)

This section shows the defect formation energies of ThO_2 obtained by DFTs and BPNNs. The defect structures such as Schottky and Frenkel pair defects were not explicitly included in the present reference data. Therefore, it is not obvious whether BPNN can reproduce the formation enegy of these defects. Here, we calculated the formation energies of Schottky, oxygen Frenkel pair, and thorium Frenkel pair defects, respectively. We used $3\times3\times3$ supercell in both calculations using DFTs and BPNNs. We employed the defect configurations of Schottky, oxygen Frenkel pair, and thorium Frenkel pair as done in the reference[7]. In the calculation of Schottky defect, we considered three types of vacancy configurations as

$$\mathrm{Sch1:}\quad \mathrm{Th_{vac}} = (0,0,0).\quad \mathrm{O_{vac,1}} = (1/4,1/4,1/4),\quad \mathrm{O_{vac,2}} = (-1/4,-1/4,-1/4), \tag{7}$$
$$\mathrm{Sch2:}\quad \mathrm{Th_{vac}} = (0,0,0).\quad \mathrm{O_{vac,1}} = (1/4,1/4,1/4),\quad \mathrm{O_{vac,2}} = (-1/4,1/4,-1/4), \tag{8}$$
$$\mathrm{Sch3:}\quad \mathrm{Th_{vac}} = (0,0,0).\quad \mathrm{O_{vac,1}} = (1/4,1/4,1/4),\quad \mathrm{O_{vac,2}} = (-1/4,1/4,-1/4), \tag{9}$$

where $\mathrm{Th_{vac}}$ and $\mathrm{O_{vac}}$ denote the positions of the thorium and oxygen vacancies on 4a and 8c site, respectively. Here, the coordinates of the defect position are represented by fractional coordinate of the unit cell of ThO_2. For the oxygen Frenkel pair, we used the defect configurations

$$\mathrm{OFP1:}\quad \mathrm{O_i} = (1/2,1/2,1/2).\quad \mathrm{O_{vac}} = (-1/4,-1/4,-1/4), \tag{10}$$
$$\mathrm{OFP2:}\quad \mathrm{O_i} = (1/2,1/2,1/2).\quad \mathrm{O_{vac}} = (-1/4,-1/4,1/4), \tag{11}$$

where $\mathrm{O_i}$ mean the position of an interstitial oxygen atom on 4b site. Finally, the thorium Frenkel pair defect energies (ThFP) were calculated for the second nearest neighbor configuration with a thorium interstitial (4b site) and vacancy (4a site). The defect formation energies obtained by DFTs and BPNNs are listed in table S5. We can find that BPNNs reproduced the defect formation energies with a certain degree of accuracy. Here, we again note that we did not explicitly include the above defect structures in the present reference data. It is considered that the inclusion of liquid structures and the structures with oxygen diffusion at high temperatures made BPNNs possible to describe the various defect structures. However, comparing the errors for the defect formation energies obtained by BPNNs, there were some differences in accuracy against DFT data. Especially, BPNN-PBEsol showed a large error

for the ThFP formation energy. The difference in accuracy for DFT data is considered due to differences in the method used to generate the reference data. The reference data set of BPNN-PBEsol was generated by only FPMD simulation, whereas these of BPNN-LDA and BPNN-SCAN were mainly created by the adiabatic learning scheme. The ranges of forces contained in the reference data of BPNN-LDA and BPNN-SCAN were broader than that of BPNN-PBEsol, as shown in figure S2. The reference data of BPNN-LDA and BPNN-SCAN contained more diverse structures than that of BPNN-PBEsol, which enable BPNN-LDA and BPNN-SCAN to predict the ThFP formation energy with a certain degree of accuracy. Of course, the explicit inclusion of the defect structures in the reference data is expected to improve further the accuracy of BPNNs for the defect formation energies.

7 Born effective charges and dielectric constants

In phonon calculation in the main text, we used the Born effective charge and the dielectric constants to calculate the non-analytical term correction to the dynamical matrix. They were obtained from the response to finite electric fields $|\boldsymbol{\mathcal{E}}| = 0.01$ (eV/Å) using VASP. The phonon dispersion curves computed by (BPNN-)LDA, (BPNN-)PBEsol, and (BPNN-)SCAN are corrected using the dipole-dipole interaction correction[8,9] with the Born effective charge and the dielectric constants obtained by LDA, PBEsol, and SCAN, respectively. The detailed values were summarized in Table S6. In phonon calculations using BD08 and cooper potentials, we used the non-analytical term correction with the Born effective charge and the dielectric constants obtained by LDA calculation.

Table S6: Born effective charges and dielectric constants.

	LDA	PBEsol	SCAN
Dielectiric constants ϵ	4.66	4.60	4.30
Born effective charges of Th (e)	5.54	5.59	5.56
Born effective charges of O (e)	−2.77	−2.80	−2.78

References

[1] Wenwen Li and Yasunobu Ando. Dependence of a cooling rate on structural and vibrational properties of amorphous silicon: A neural network potential-based molecular dynamics study. *The Journal of Chemical Physics*, Vol. 151, No. 11, p. 114101, 2019.

[2] Matti Hellström and Jörg Behler. Structure of aqueous naoh solutions: Insights from neural-network-based molecular dynamics simulations. *Physical Chemistry Chemical Physics*, Vol. 19, No. 1, pp. 82–96, 2017.

[3] Andreas Singraber, Jörg Behler, and Christoph Dellago. Library-based lammps implementation of high-dimensional neural network potentials. *Journal of Chemical Theory and Computation*, Vol. 15, No. 3, pp. 1827–1840, 2019.

[4] Andreas Singraber, Tobias Morawietz, Jörg Behler, and Christoph Dellago. Parallel multistream training of high-dimensional neural network potentials. *Journal of Chemical Theory and Computation*, Vol. 15, No. 5, pp. 3075–3092, 2019.

[5] Keita Kobayashi, Yuki Nagai, Mitsuhiro Itakura, and Motoyuki Shiga. Self-learning hybrid monte carlo method for isothermal-isobaric ensemble: Application to liquid silica. *The Journal of Chemical Physics*, Vol. 155, No. 3, p. 034106, 2021.

[6] S.I. Potashnikov, A.S. Boyarchenkov, K.A. Nekrasov, and A.Ya. Kupryazhkin. High-precision molecular dynamics simulation of uo2-puo2: Pair potentials comparison in uo2. *Journal of Nuclear Materials*, Vol. 419, No. 1, pp. 217–225, 2011.

[7] K. Govers, S. Lemehov, M. Hou, and M. Verwerft. Comparison of interatomic potentials for uo2. part i: Static calculations. *Journal of Nuclear Materials*, Vol. 366, No. 1, pp. 161–177, 2007.

[8] X. Gonze, J.-C. Charlier, D.C. Allan, and M.P. Teter. Interatomic force constants from first principles: The case of α-quartz. *Phys. Rev. B*, Vol. 50, pp. 13035–13038, Nov 1994.

[9] Xavier Gonze and Changyol Lee. Dynamical matrices, born effective charges, dielectric permittivity tensors, and interatomic force constants from density-functional perturbation theory. *Phys. Rev. B*, Vol. 55, pp. 10355–10368, Apr 1997.
